# A zebrafish forward genetic screen identifies an indispensable threonine residue in the kinase domain of PRKD2

**DOI:** 10.1242/bio.058542

**Published:** 2021-03-09

**Authors:** Panagiota Giardoglou, Despina Bournele, Misun Park, Stavroula Kanoni, George V. Dedoussis, Susan F. Steinberg, Panos Deloukas, Dimitris Beis

**Affiliations:** 1 Zebrafish Disease Model lab, Biomedical Research Foundation Academy of Athens, Athens 115 27, Greece; 2 Department of Nutrition and Dietetics, School of Health Science and Education, Harokopio University of Athens, Athens 176 71, Greece; 3 Department of Pharmacology, Columbia University, New York 100 27, USA; 4 William Harvey Research Institute, Barts and The London School of Medicine and Dentistry, Clinical Pharmacology Centre, Queen Mary University of London, London, EC1M 6BQ, UK; 5 Princess Al-Jawhara Al-Brahim Centre of Excellence in Research of Hereditary Disorders (PACER-HD), King Abdulaziz University, Jeddah 222 52, Saudi Arabia

**Keywords:** Protein kinase D2, Cardiovascular development, Cardiac valves, Zebrafish

## Abstract

Protein kinase D2 belongs to a family of evolutionarily conserved enzymes regulating several biological processes. In a forward genetic screen for zebrafish cardiovascular mutants, we identified a mutation in the *prkd2* gene. Homozygous mutant embryos develop as wild type up to 36 h post-fertilization and initiate blood flow, but fail to maintain it, resulting in a complete outflow tract stenosis. We identified a mutation in the *prkd2* gene that results in a T757A substitution at a conserved residue in the kinase domain activation loop (T714A in human PRKD2) that disrupts catalytic activity and drives this phenotype. Homozygous mutants survive without circulation for several days, allowing us to study the extreme phenotype of no intracardiac flow, in the background of a functional heart. We show dysregulation of atrioventricular and outflow tract markers in the mutants and higher sensitivity to the Calcineurin inhibitor, Cyclosporin A. Finally we identify TBX5 as a potential regulator of PRKD2. Our results implicate PRKD2 catalytic activity in outflow tract development in zebrafish.

This article has an associated First Person interview with the first author of the paper.

## Abstract (precis)

**Summary:** We identified, through a zebrafish forward screen, an evolutionarily conserved residue in the catalytic domain of protein kinase D2 and its homologues.

## INTRODUCTION

Some form of congenital heart disease (CHD) complicates up to 20 out of 1000 live births, with septal and valve defects being the most common form of CHD (Hoffman and Kaplan, 2002), providing a strong rational for studies that unravel fundamental mechanisms of cardiac morphogenesis and valve formation. To this end, various animal models that recapitulate human heart diseases associated with valve defects have been generated. Many studies have focused on atrioventricular (AV) endocardial cushion development, a milestone in cardiac valve formation. While this developmental stage has been studied extensively in mouse and chicken models, several aspects of heart formation are evolutionarily conserved among different classes of vertebrates including in zebrafish (Beis et al., 2015), a model that is highly genetically homologous to human and is uniquely suited for this type of analysis; zebrafish are amenable to non-invasive imaging techniques as well as forward and reverse genetic manipulations. While proper cardiac valve development and morphogenesis depends on the shear-stress imposed on endocardial cells at the valve forming regions as a result of intracardiac flow dynamics (Vermot et al., 2009; Kalogirou et al., 2014), the absence of circulation does not present an obstacle for these studies in zebrafish, since embryo development is maintained via oxygen delivery through passive diffusion (reviewed in Bournele and Beis, 2016; Giardoglou and Beis, 2019). Not surprisingly, mutants identified in zebrafish with intracardiac flow defects also exhibit valve morphogenesis defects with the most representative being the *silent heart* (*sih*) mutant. *sih* carry a mutation in the cardiac troponin T gene (Tnnt2) causing a total absence of cardiac contractility. While the embryos survive for several days and appear morphologically wild type, both the AV as well as outflow tract valves remain severely hypoplastic (Sehnert et al., 2002; Bartman et al., 2004).

The zebrafish heart has two chambers, a single atrium and a single ventricle. Blood flow enters the atrium through the sinus venosus and passes through the AV canal to the ventricle and then exits through an outflow tract consisting of the bulbus arteriosus and the ventral aorta. The AV valve and outflow tract valve prevent blood regurgitation with the AV region expressing differentiation markers at ∼37 h post fertilization (hpf) (Walsh and Stainier, 2001). The first step in valve formation is the generation of endocardial cushions (EC), transient structures that result from expansion of the extracellular matrix (ECM) between the endocardium and myocardium at the AV canal (Beis et al., 2005). Valve endocardial cells migrate into this region and undergo endothelial-to-mesenchymal transition (EMT), becoming valve interstitial cells (VICs).

Studies to date implicate Nfatc1 signaling (Sugi et al., 2004; Timmerman et al., 2004; Gunawan et al., 2020), Notch1 and bone morphogenetic protein 2 and 4 (BMP2/4) signaling pathways (de la Pompa et al., 1998; Chang et al., 2004) and Calcineurin/NFAT-dependent suppression of vascular endothelial growth factor (*vegf*) as mechanisms that regulate AV canal differentiation, EC formation, and/or EMT (de la Pompa et al., 1998; Chang et al., 2004; Gunawan et al., 2020). While there is evidence that protein kinase D (PRKD) family enzymes are expressed in cardiac valves (Oster et al., 2006) and they contribute to the regulation of a histone deacetylase 5 (HDAC5)-dependent pathway that regulates Notch expression (Just et al., 2011), their precise role in the control of AV valve formation remains uncertain.

PRKD consists of a family of evolutionarily conserved signal-activated enzymes (PRKD1, PRKD2, and PRKD3) (Valverde et al., 1994; Hayashi et al., 1999; Sturany et al., 2001) that play critical roles in fundamental biological processes (Manning et al., 2002) and contribute to the pathogenesis of a large number of clinically important diseases, including pancreatitis (Piscuoglio et al., 2016), various cancers (Yuan and Pandol, 2016), human heart failure development and cardiac hypertrophy (Wei et al., 2015). PRKD isoforms share a modular domain structure consisting of a C-terminal kinase domain and an N-terminal regulatory domain carrying tandem C1A/C1B motifs that anchor full-length PRKD to diacylglycerol-/phorbol ester-containing membranes (Iglesias et al., 1998; Iglesias and Rozengurt, 1999; Rey and Rozengurt, 2001; Chen et al., 2008) and a pleckstrin homology motif that participates in intramolecular auto-inhibitory interactions (Iglesias and Rozengurt, 1998; Waldron et al., 1999). PRKD isoform activation is generally attributed to growth factor-dependent mechanisms that promote diacylglycerol accumulation and protein kinase C- (PKC-) dependent trans-phosphorylation of PRKD at serine residues in the activation loop, a highly conserved 20–30 residue flexible segment in kinase domain that sits near the entrance to the active site and functions to structure the enzyme for catalysis (Rozengurt et al., 2005). Activated PRKD1 and PRKD2 then autophosphorylate at a serine residue in a PDZ domain-binding motif/PRKD consensus phosphorylation motif at the extreme C-terminus. PRKD3 lacks this autophosphorylation site.

While cardiac tissues co-express PRKD1, PRKD2, and PRKD3, and previous studies show that PRKD isoforms can be activated in a stimulus-specific manner in cardiomyocytes (Guo et al., 2011), most studies have focused on the cardiac actions of PRKD1, which is downregulated during normal postnatal development, upregulated in various cardiac hypertrophy/failure models, and contributes to adverse cardiac remodeling and has been implicated in syndromic congenital heart defects (Speliotes et al., 2010; Johnson et al., 2015). Shaheen et al. identified a homozygous truncating mutation in *PRKD1* that leads to the generation of a catalytically inactive protein (that contains the entire N-terminal regulatory domain but only the first 35 residues at the N-terminus of the kinase domain) in patients with truncus arteriosus (Shaheen et al., 2015). Studies from Sifrim et al. (Sifrim et al., 2016) and more recently Alter et al. (Alter et al., 2020) identify heterozygous *de novo* missense mutations in *PRKD1* in five patients with syndromic congenital heart disease. Of note, three of these patients had identical inactivating missense Gly592Arg mutations in the PRKD1 kinase domain.

Studies of the role of PRKD2 in cardiac function and angiogenesis are more limited, but there is some evidence that defective PRKD2 signaling contributes to the development of human hypertrophic cardiomyopathy (Tsybouleva et al., 2004) and PRKD2 activation of a HDAC5 signaling pathway controls the expression of genes involved in Notch signaling during valve formation in zebrafish embryos (Just et al., 2011). This study uses a zebrafish forward genetic screen (Beis et al., 2005) to identify a mutant line that carries an A to G mutation resulting in a T757A substitution of Prkd2 (T714A in PRKD2) that disrupts catalytic activity and results in outflow tract stenosis in zebrafish embryos by 72 hpf.

## RESULTS

### Impaired heart development in *s411* mutants

**Fig. 1:**
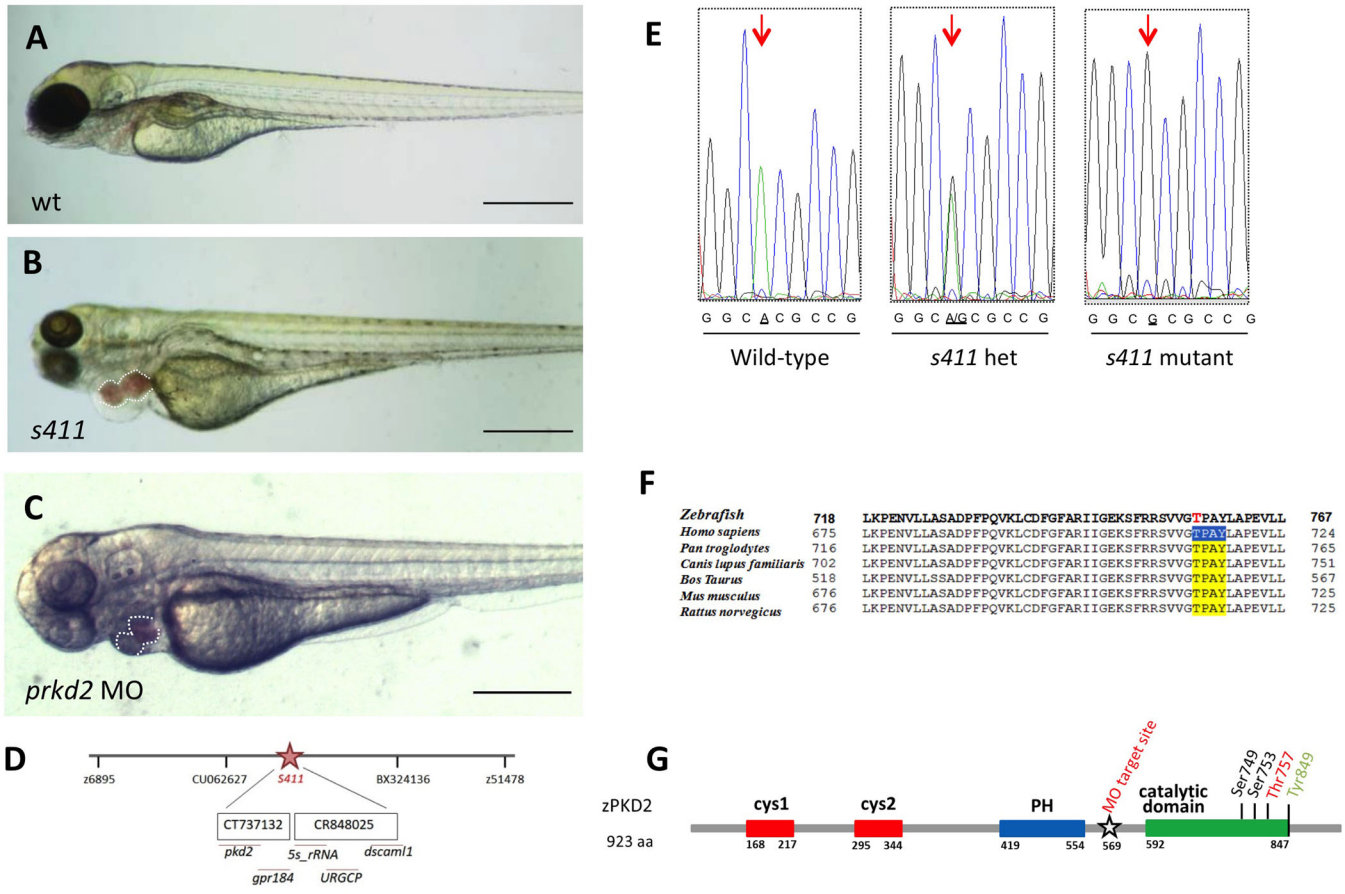
**. *s411* carry a mutation in the *prkd2* gene.** (A,B) Bright field image analysis of a wild-type zebrafish embryo compared to an *s411* mutant embryo at 72 hpf. Heterozygous adults that carry the *s411* recessive mutation give 25% offspring mutant embryos exhibiting heart edema, inadequate blood circulation leading to complete outflow tract stenosis and blood regurgitation by 72 hpf. Scale bars: 500 μm. (C) Bright-field image analysis *prkd2*-targeted morpholino-injected embryo at 72 hpf. MO-*prkd2*-injected embryos resemble the *s411* phenotype possessing the same features as *s411* mutants (three replicates, *n*>50). Scale bar: 200 μm. (D) Bulked segregant analysis and further genetic mapping positioned the *s411* mutation on chromosome 15. Recombination analysis on 865 embryos, positioned the mutation initially between the markers, z6895 and z51478 and further fine-mapping positioned the mutation between markers we developed in the overlapping BACs: CU062627 and BX924136. (E) The *s411* embryos carry an A to G mutation that translates to a Threonine (T) to Alanine (A) amino acid change. (F) Blast analysis showed that the threonine T757 (T714 in humans) is a conserved amino acid at a highly conserved region of the C-terminus PRKD2 kinase, between several species. (G) Schematic representation of zebrafish Prkd2 kinase. It consists of 923 amino acids and the main domains of the enzyme are indicating: two cysteine-rich motif domains (cys1 and cys2), a pleckstrin homology domain (PH), and the C-terminal catalytic domain where the PKC-phosphorylation sites, Ser749 and Ser753 reside. It is also highlighted the position of *s411* mutation (A to G), a previously identified zebrafish mutation with a similar phenotype (Y849) and the position of premature stop codon after MO injection resulting to defective splicing.

The zebrafish *s411* mutant was identified during a large-scale zebrafish ENU-mutagenesis forward genetics screen (Beis et al., 2005). *s411* is a recessive mutation that results in embryonic lethality by 120 hpf. *s411* mutants initially appear morphologically as wild type, but they show a reduced circulation and a heart-specific phenotype at 48 hpf, which becomes progressively more severe (Fig. 1A,B). Specifically, *s411* mutants develop outflow tract stenosis by 72 hpf that leads to retrograde blood flow from the ventricle to the atrium and disruption of blood circulation to the rest of the embryo body (Movie 1). As a result, a severe pericardial edema starts developing from 72 hpf.

### The *s411* mutants carry T757A mutation in the *prkd2* gene

We performed mapping by chromosome walking, based on microsatellite markers and genetic polymorphism maps in order to identify the recessive mutation that results in the *s411* phenotype. Bulked segregant analysis using a panel of markers across the zebrafish genome, allowed us to map the mutation to chromosome 15 (linkage group 15). Polymorphism marker analyses of individual mutants allowed us to genetically narrow the *s411* mutation to a region between z6895 and *z51478*. Further, fine-mapping placed this mutation at a site between markers, CU062627 and BX924136 – a region that contains the candidate genes *prkd2*, *gpr184*, *5s rRNA*, *urgcp* and *dscam-like1* (Fig. 1D). To identify the mutated gene, we sequenced *s411* mutant and wild-type (wt) embryos for the genes mentioned above and identified a mutation in the coding region of *prkd2*. Zebrafish *prkd2* consists of 18 exons, encoding for a 923aa protein. The *s411* mutation causes an adenine to guanine conversion, and the substitution of a conserved Threonine to Alanine at position 757, in the Prkd2 catalytic domain (corresponding to Thr714 in human *PRKD2*; Fig. 1E,F). This threonine, located in the kinase domain activation loop, is evolutionarily conserved across *PRKD2* homologs from zebrafish to rodent and human. It is also conserved in the catalytic domain of other PRKD homologues (PRKD1 and PRKD3) and other AGC kinases (including PKA, all PKC isoforms, and AKT). These findings suggested that a Thr757Ala substitution in the Prkd2 catalytic domain might contribute to the *s411* mutant phenotype.

In order to confirm that the impaired heart development of *s411* mutants derives from the T757A mutation of Prkd2, we injected a *prkd2*-targeting morpholino at the one-cell stage of wild-type embryos to block the *prkd2* mRNA splicing. Morpholino was designed to impair the splicing site of exon 11 with the following intron. Accordingly, the injected embryos display similar phenotype to *s411* mutants at a rate of 80% (Fig. 1C). They exhibited outflow blood stenosis and blood regurgitation phenocopying s411 homozygous mutants, without developing any other morphological defects. When the cDNA of the injected embryos was sequenced, we verified that exon 11 (53 bp) was removed from the final transcript resulting in and out of frame protein following exon10 and a premature stop codon. This structure corresponds to a truncated protein that entirely misses the catalytic site of the enzyme (Fig. 1G). In addition, a previously identified mutation in Tyrosine 849 shows a similar phenotype (Fig. 1G) (Just et al., 2011).

### The T757A substitution disrupts PRKD2 kinase activity

**Fig. 2.**
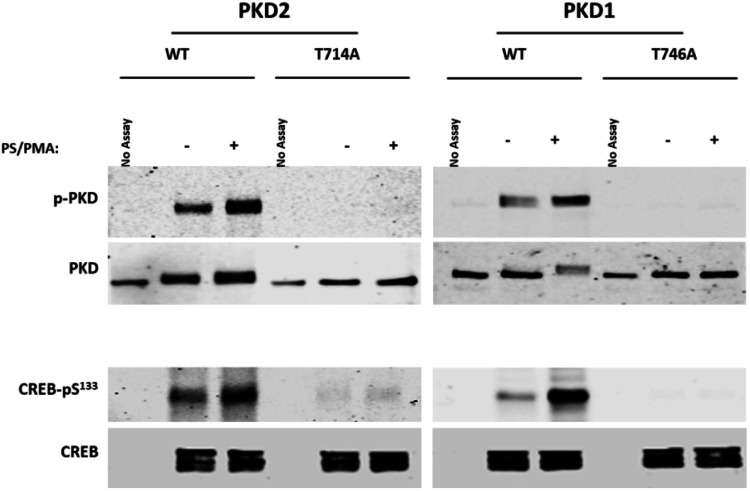
**The T757A substitution disrupts PRKD2 kinase activity.** Lysates from HEK293 cells that heterologously overexpress wild-type or mutant forms of PKD1 and PKD2 were subjected to *in vitro* immuno-complex kinase assays (IVKAs) in the absence or presence of PS/PMA and immunoblot analysis was used to track PKD C-tail autophosphorylation as well as PKD1 phosphorylation of recombinant CREB (added as a heterologous substrate). All results were replicated in three separate experiments.

We performed *in vitro* kinase assays (IVKAs) to examine the functional consequences of the threonine-to-alanine substitution in the activation loop of Prkd2. Fig. 2 shows that WT-PRKD2 is recovered from HEK293 cells with a low level of anti-PRKD1-pSer^910^ immunoreactivity and CREB-Ser^133^ kinase activity and that WT-PRKD2 autocatalytic and CREB kinase activities increase in response to treatment with PS/PMA (a pharmacologic activator of PRKD enzymes). In contrast, PRKD2 harboring an activation loop T757A substitution is catalytically inactive; it does not autophosphorylate at its C-terminal autophosphorylation site and it shows no CREB kinase activity (no activity toward a heterologous substrate). Of note, threonine-to-alanine substitution at the cognate site in PRKD1 results in the identical phenotype – it renders the enzyme catalytically inactive.

### *s411* mutants exhibit defects in cardiac morphogenesis and ectopic expression of AV markers

**Fig. 3.**
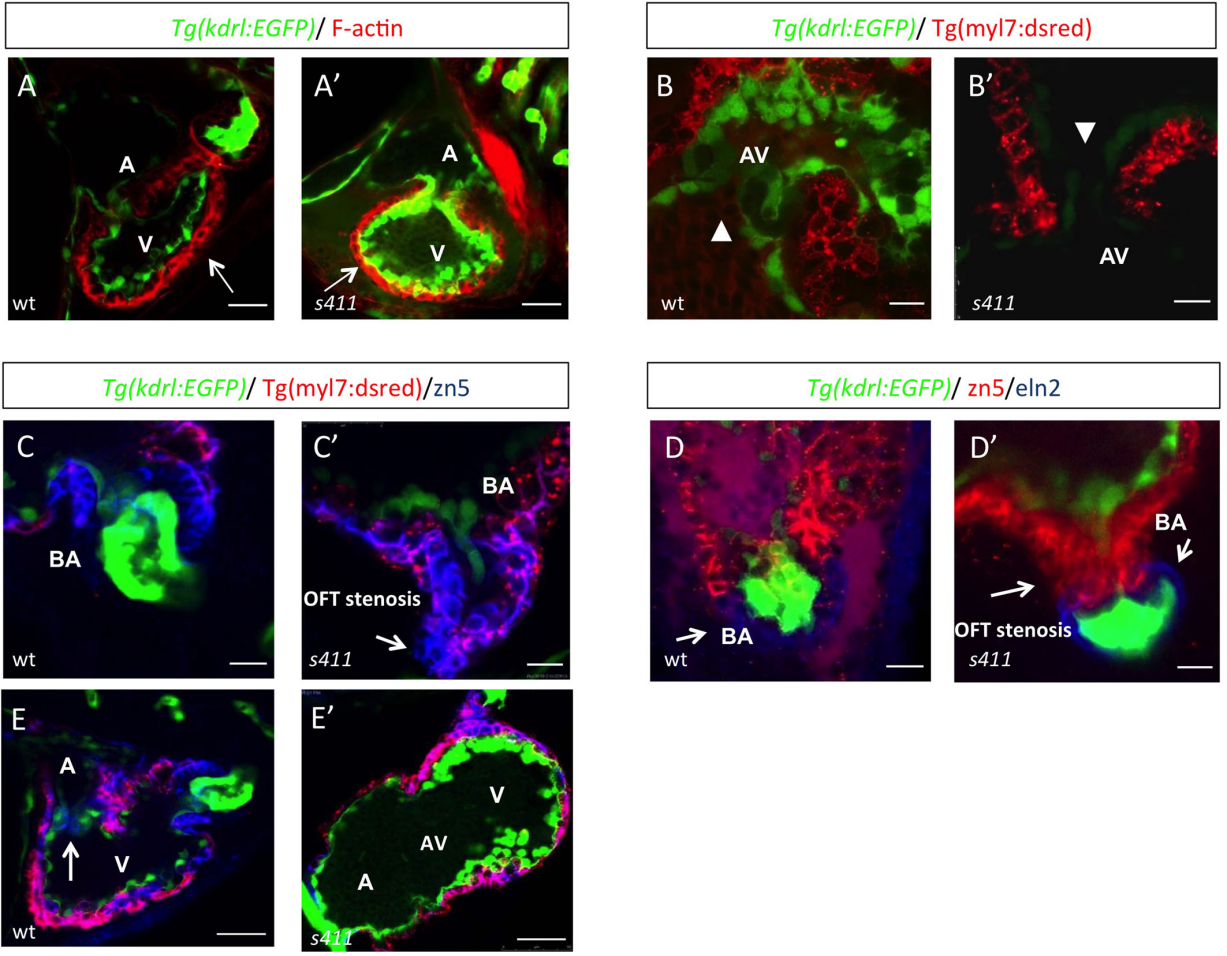
***s411* embryos show aortic valve stenosis.** (see also Movies 2 and 3). Confocal images of the heart (A,A′,E,E′), the AV canal (B,B′), the outflow track (C,C′) and the bulbus arteriosus (D,D′) at 72 hpf. *Tg(kdrl::EGFP)* (green) embryos were stained for F-actin (red) (A,A′) or zn5 (pseudo-colored red) and eln2 (pseudo-colored blue) (D,D′). *Tg(kdrl::EGFP) (green)/Tg(myl7:DsRed)* embryos were stained for zn5 (pseudo-colored blue) (C,C′,E,E′). (A,A′) In *s411* embryos, the myocardium appears thinner compared to the wild-type embryos. Arrows indicate the myocardial wall. (B,B′) Endocardial cells residing at AV canal extend in two layers at wild-type embryos whereas the *s411* mutants obtain a single-layer of AV endocardial cells. Arrowheads indicate the endocardial cells of AV. (C,C′) The endocardial cells of outflow track are in tight contact in *s411* mutant embryos. This structural feature leads to stenosis formation. Arrow indicates the junction between the neighboring endocardial cells. (D,D′) Bulbus arteriosus consists of endocardial cells surrounded by a layer of smooth muscle cells. *s411* mutants have a blocked bulbus arteriosus. Arrows show the elastin-positive cells and the stenosis of mutant embryos. (E,E′) In *s411* embryos, the endocardial monolayer of AV cells does not express zn5 compared to the two-layer zn5^+^ AV endocardial cells in wild-type embryos, which indicates the malformation of atrioventricular cardiac valve during development. A, atrium; V, ventricle; AV, atrioventricular, BA, bulbus arteriosus. *n*=10 in each of three independent experiments. Scale bars: 50 μm for A,A′, E,E′ and 20 μm for B–D′.

To dissect at the cellular level the phenotypic features of the *prkd2* hearts, we performed immunohistochemistry analyses. Using rhodamine phalloidin (filamentous actin staining) and *Τg(myl7:dsred)* we showed that the ventricular myocardium of *prkd2^s411^* appears to be much thinner at 72 hpf compared to wild-type siblings (Fig. 3A,A′,B,B′). In addition, we observed that there is a single layer of cuboid AV endocardial cells in mutants whereas wild-type embryos appear to have two layers of cuboid cells at the AV canal (Fig. 3B,B′). At the outflow tract, there is a complete stenosis occurring at 72 hpf (3C,C′), which is the earliest defect we can detect (Movies 2 and 3). This blocks the circulation towards the branchial vasculature at the level of the intersection between the ventricle and the bulbus arteriosus. Specifically, endocardial cells of this area have collapsed disturbing the continuation of the endocardium to endothelial cells (Fig. 3D,D′). In addition, the single layer of cuboidal cells of atrioventricular valve cells in *prkd2^s411^* does not express zn5 – a cell–cell adhesion marker that is localized in myocardial cells and differentiated AV canal cells – in contrast to wild-type embryos (Fig. 3E,E′). These data confirm that the outflow tract stenosis and blood regurgitation also affects the morphogenesis of the atrioventricular cardiac valves during development, also as a consequence of the intracardiac flow dynamic breakdown.

**Fig. 4.**
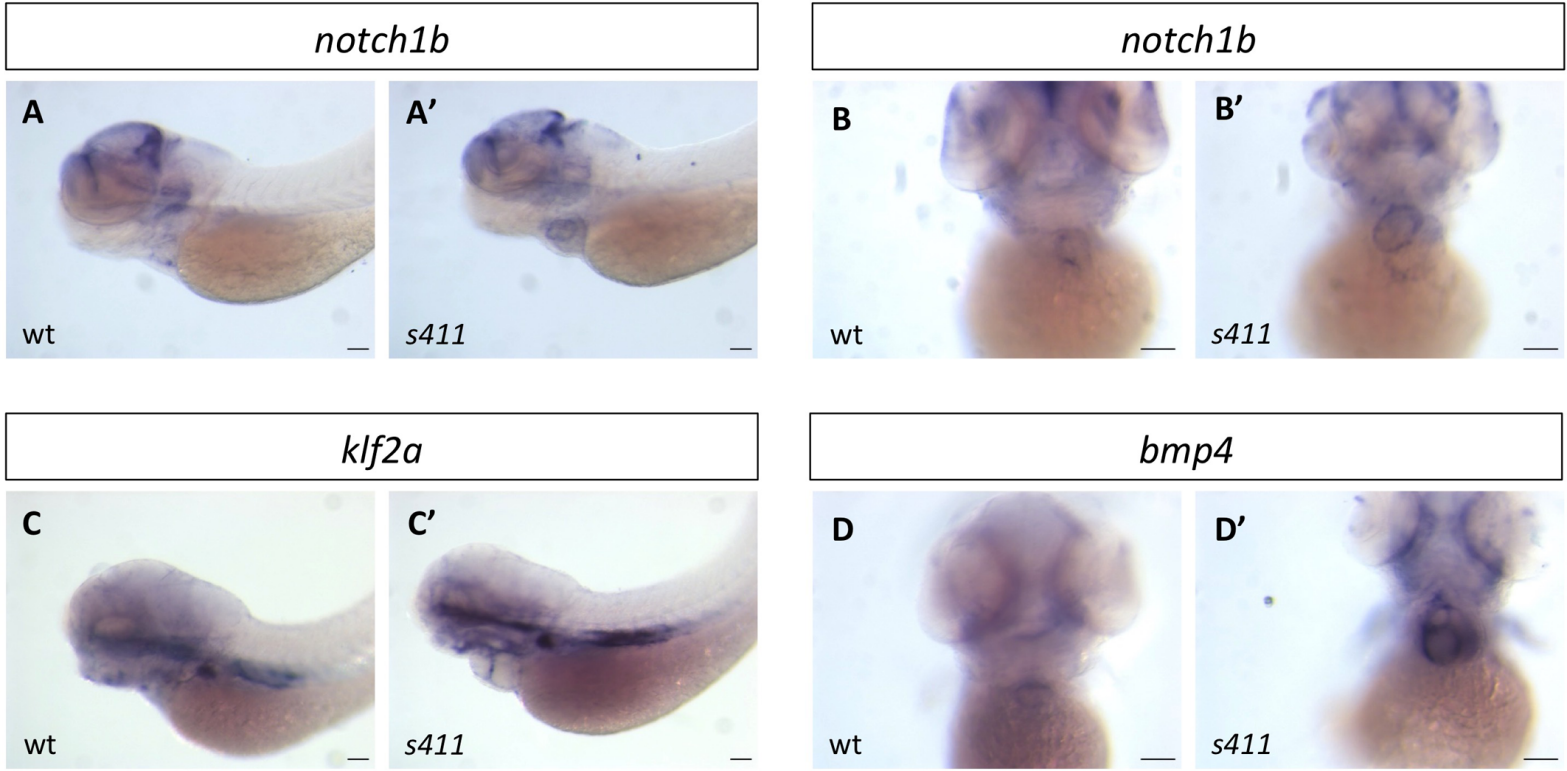
**Atrioventricular canal markers remain expressed throughout the heart in *s411* mutant embryos.** Ectopic expression of atrioventricular canal (AV) markers in *s411* mutant embryos as shown by wholemount *in situ* hybridization. *notch1b* antisense probe in lateral (A,B) and dorsal (A′,B′) view of wild-type embryos (A,B) and *s411* mutants, respectively (A′,B′), at 72 hpf. *klf2a* in wild-type (C) and *s411* mutants (C′) and *bmp4* in wild-type (D) and *s411* mutants (D′) at 72 hpf. All three AV differentiation markers show restricted expression pattern in the AV and outflow tract of wild-type embryos, while they remain expressed throughout the heart in *s411* mutants. *n*=10 in each of three independent experiments. Scale bars: 150 μm.

**Fig. 5.**
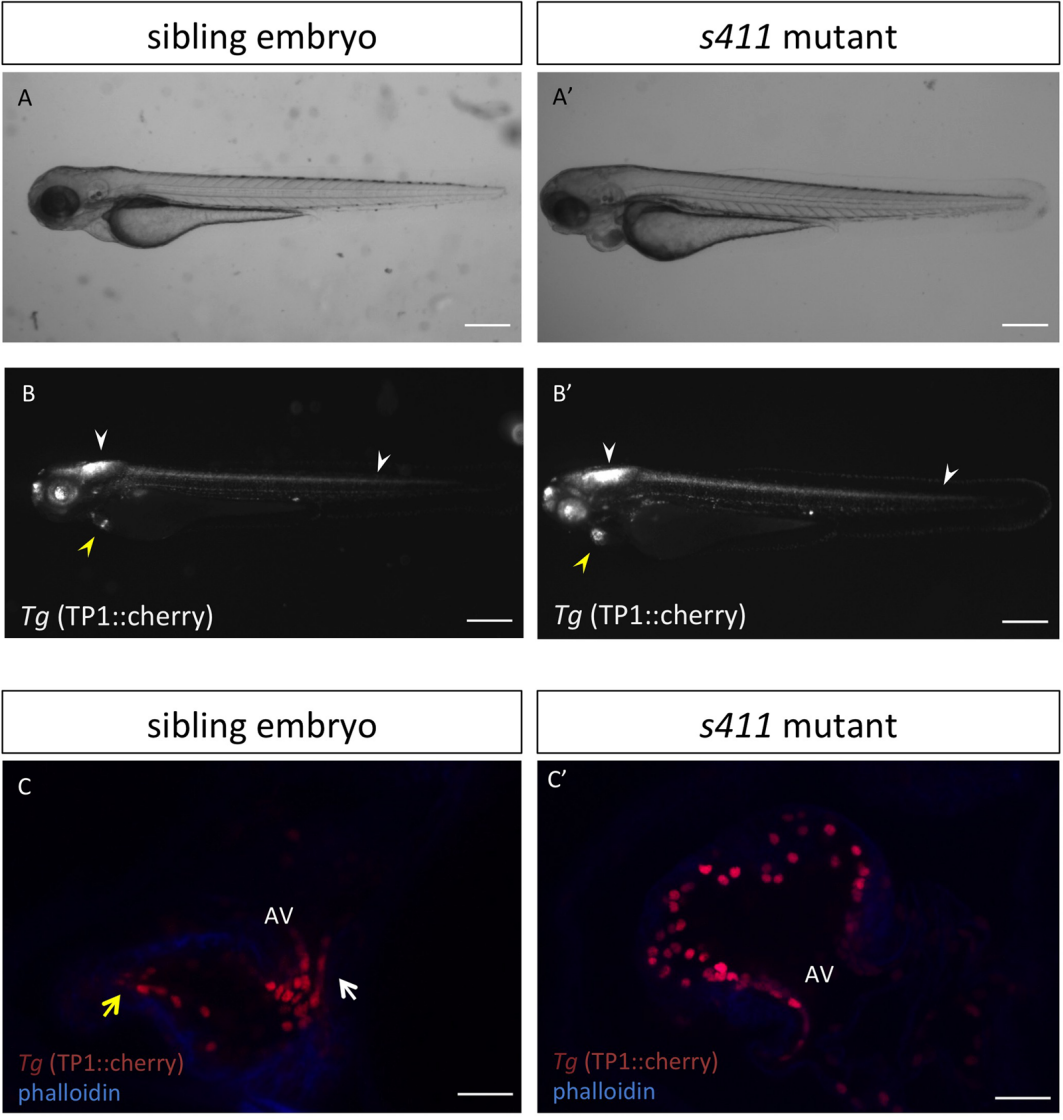
**Ectopic activation of endocardial Notch signaling in *s411* embryos carrying the T757A PRKD2 mutation.** Bright field (A,A′) and fluorescence (B,B′) analysis of *s411* sibling and mutant embryos carrying the *Tg(Tp1:mCherry)* (pseudo-colored grey). Scale bars: 500 μm. Confocal analysis of 150 μm cardiac slices of *s411* sibling and mutant embryos carrying the *Tg(Tp1:mCherry)* co-stained with 633-phalloidin (blue) (C,C′); Scale bar: 25 μm. Notch signaling is active in several tissues and organs throughout the embryo and restricted to the AV canal and OFT of wild-type embryos at 72 hpf (B,C). In *s411* mutants, Notch signaling appears unaffected in the embryo (B′, white arrowheads) but remains active throughout the ventricular endocardium (B′, yellow arrowheads, C′). White and yellow arrows (C) indicate the Notch-positive cells at AV canal and OFT, respectively. AV, atrioventricular; OFT, outflow tract. *n*=10 in each of three independent experiments.

We also performed *in situ* hybridization of *notch1*, *bmp4* and *klf2a*. The expression of these genes is initially throughout the heart and gets restricted to the AV canal cells by 72 hpf. These are the first hallmarks of AV differentiation, and a reliable indicator of proper valve development (Stainier et al., 2002; Beis et al., 2005). All the three markers (*notch1b*, *klf2a*, and *bmp4*) remain ectopically expressed throughout the heart of *s411* mutants (Fig. 4A′–D′, compare with A–D). In addition, *s411* mutants carrying the *Tg(TP1:mCherry)* (a transgenic reporter line used as a biomarker for Notch signaling activation) show ectopic activation throughout the ventricular endocardium (Fig. 5C′, compare to 5C). This confirms the *in situ* data as well as previous reports that Prkd2 controls Notch signaling via HDAC regulation. Notably, Notch signaling remains unaffected in non-cardiac tissue (Fig. 5B′, compare with B). These results reveal the critical role of Prkd2 in cardiac valve morphogenesis during early development.

### PRKD2 and Calcineurin (CN) cooperate to regulate heart development

**Fig. 6.**
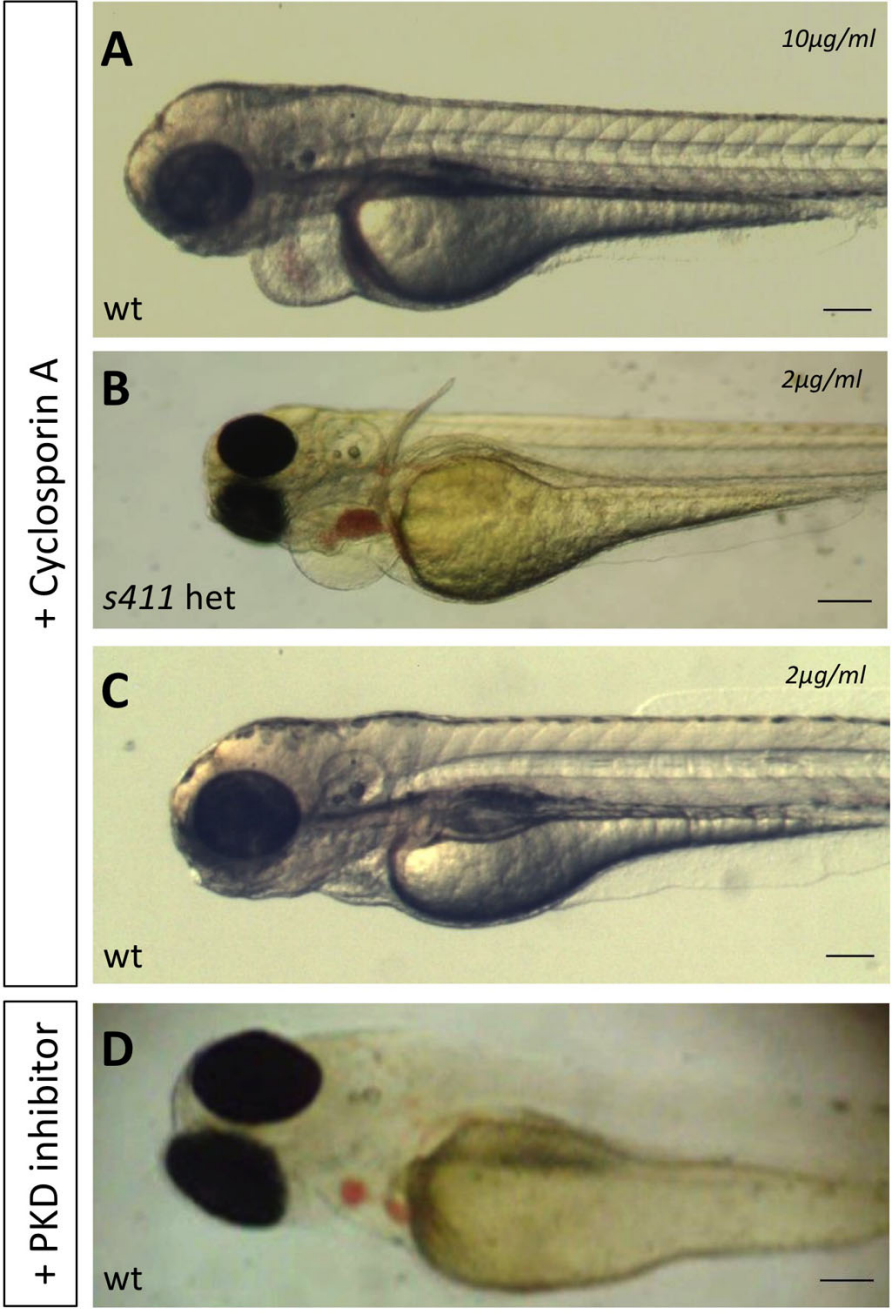
**Disruption of calcineurin/NFATc signaling and blocking PRKD activity exhibits a similar phenotype to *s411* mutant embryos.** Incubation of wild type (A) with 10 μg ml^−1^ cyclosporine A (CsA) results to a *s411* phenotype. *s411* heterozygous (B) embryos treated with cyclosporine A, at 2 μg ml^−1^, a sublethal dose of CsA show phenotype while there is no effect in wild-type embryos (C) when treated at this dose. (D) Treatment of wild-type embryos with a protein kinase D family inAhibitor, 2,3,4,5 -Tetrahydro-7-hydroxy- 1H-benzofuro[2,3-c]azepin-1-one between 30–72 hpf phenocopies *s411* mutants showing that this is the critical window of *prkd2* activity in the heart. *n*=20 in each of three independent experiments. Scale bars: 100 μm.

The Calcineurin/Nuclear Factor of Activated T-cells (CN/NFATc) signaling pathway plays a crucial role in cardiac valve morphogenesis (de la Pompa et al., 1998). Studies in embryonic mouse and fish heart valve remodeling link this pathway to the production of soluble factors such as vascular endothelial growth factor (VEGF) that regulate EMT (Timmerman et al., 2004; Gunawan et al., 2020). Since NFAT enters the nucleus and drives the transcription of genes involved in heart formation only when dephosphorylated, NFAT activation can result from either enhanced dephosphorylation (by activated calcineurin) or reduced phosphorylation (by a PKC/PRKD-dependent mechanism) (Prasad and Inesi, 2009). Wild-type and heterozygous *s411* mutant zebrafish embryos were treated with Cyclosporine (CsA), a Calcineurin inhibitor (under conditions that abrogate CN activity) to test the hypothesis that CN and PRKD2 cooperate to regulate zebrafish valve development. Fig. 6A shows that wild-type CsA-treated embryos display heart defects nearly identical to those identified in *s411* mutant embryos. Interestingly, heterozygous *s411* embryos from an heterozygous cross showed enhanced sensitivity to CsA, displaying a phenotype at a CsA dose that does not affect wild-type embryos (2 μg ml^−1^) (Fig. 6B,C). The observation that CsA treatment of wild-type embryos recapitulates the *s411* Prkd2 T757A phenotype suggests that CN and PRKD2 act in a reciprocal manner to control the phosphorylation of signaling proteins that regulate valve development.

Finally, wild-type zebrafish embryos were treated with CID755673, an inhibitor of PRKD activity. While early treatment with the inhibitor led to severely dysmorphic embryos (implicating PRKD activity in overall embryo development), CID755673 treatment initiated at 30 hpf resulted in the appearance of embryos that phenocopied the *s411* morphology (outflow blood stenosis and blood regurgitation), (Fig. 6D compare to A). These findings identify a specific time-window in which PRKD activity is required for cardiac development in zebrafish.

### Translational implications for a Tbx5-Prkd2 axis

PRKD2 is significantly associated with coronary heart disease, but does not reach the genome-wide threshold. It is interesting to note that the associated variant rs425105 is an eQTL for PRKD2 in various human tissues.

**Fig. 7.**
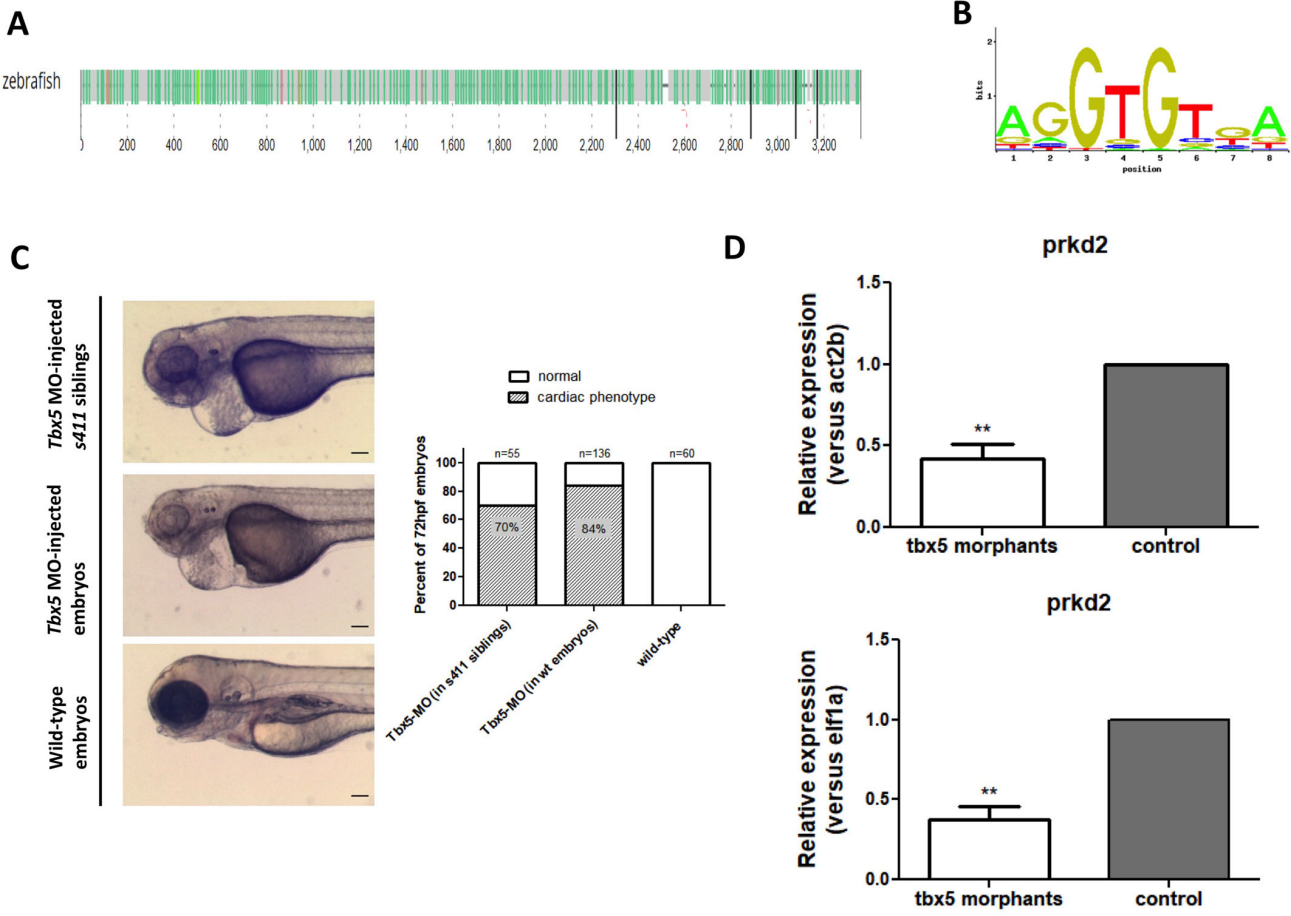
**PRKD2 association with TBX5a regulation.** Multiple predicted transcription factor Tbx5a binding sites in the promoter region of zebrafish Prkd2 via the ConTraV3 web server (A) and the possible DNA binding motif (B). Brightfield image analysis and quantification of 72 hpf *tbx5a*-morpholino injected *s411* siblings, *tbx5a*-morpholino injected wild-type and control wild-type embryos (C). *tbx5*-morpholino injected *s411* mutant embryos exhibit an earlier more severe phenotype reminiscent to the *tbx5* morphants and mutants. Scale bars: 100 μm. As measured via rt-qPCR, *prkd2* expression levels in *tbx5* morphants are reduced compared to uninjected siblings at 56 hpf, normalized both to *act2b* and *elf1a* as reference genes (D). *n*=6 independent replicates, 15 larvae per sample, Student's *t*-test (two-tailed distribution, paired), ** significantly different *P*-value <0.01, error bars +s.e.m.

There are two SNPs in perfect LD in the region (r2=1) rs425105 and rs60652743. The latter, rs60652743, https://pubs.broadinstitute.org/mammals/haploreg/detail_v4.1.php?query=&id=rs60652743 may be of interest as it will affect TBX5 binding sites. *Tbx5* encodes for a T-box containing transcription factor (TF), which plays a pivotal role in heart, eyes and forelimb development in many vertebrate species (Chapman et al., 1996; Gibson-Brown et al., 1998; Begemann and Ingham, 2000). Homozygous mutation of *tbx5a* in zebrafish embryos leads to lethal cardiac looping defects and impairment of fin initiation and morphogenesis (also known as *heartstrings*) (Garrity et al., 2002). In order to evaluate the potential functional association between *tbx5a* and *prkd2*, we first examined whether *prkd2* area in zebrafish contains Tbx5a TF binding sites (TFBS) using the Bio-tool of TFBS identification across species, ConTra v3 (Kreft et al., 2017). This database visualizes and identifies TFBS in any region surrounding a gene of interest. An analysis of a promoter region set 3000 bp upstream and 300 bp downstream of the first exon of *prkd2* identified *s*everal sites correspond to Tbx5a DNA binding motifs (Fig. 7A,B). Moreover, *tbx5a* morpholino injected embryos showed an earlier and more severe phenotype also in a *s411* heterozygous incross, with *tbx5a* morphants exhibiting a failure of heart looping and absence of pectoral fin budding (Fig. 7C, with the cardiac defects appearing earlier and being more severe when compared to the *s411* mutants) and reduced *prkd2* expression (Fig. 7D). These results are consistent with the notion that Tbx5a regulates *prkd2* expression.

## DISCUSSION

Heart valve defects are a leading cause of CHD, providing a strong rationale for studies that dissect the genetic and molecular factors underlying heart valve formation. Zebrafish embryos survive early stages of development without a functional cardiovascular system and therefore provide a particularly useful model to elucidate mechanisms of valve formation. This study uses a zebrafish model to identify the *s411* mutant (that harbors a T757A substitution that inactivates Prkd2) with a complete outflow tract stenosis.

Previous studies implicate a Prkd2-HDAC5 pathway in valve formation (Just et al., 2011). However, the observation that BMP signaling is impaired in *s411* mutants and that *s411* heterozygous embryos are hypersensitive to sublethal concentrations of the Calcineurin inhibitor cyclosporine would suggest that PKD regulates additional signaling pathways that control AV specification. Both PRKD1 and PRKD3 have been reported to exhibit a synergistic effect with the calcineurin signaling pathway to promote the expression of specific genes (Kim et al., 2008; Li et al., 2011). Although, it was demonstrated *in vivo* that PRKD1 stimulates myocyte enhancer factor-2 (MEF2) activity and it is involved in pathological cardiac remodeling in mice (Fielitz et al., 2008), it was then studied in skeletal muscle and a possible mechanism through which PRKD1 co-operates with calcineurin to drive MEF2 expression and slow-twitch fiber phenotype was proposed (Kim et al., 2008). In addition, another study showed that PRKD3 is required for the NFATc4, Nkx2.5, and GATA4 expression while acting downstream of calcineurin-activated NFATc1 and c3 in pathological cardiac hypertrophy (PCH) model (Li et al., 2011). Therefore, the signaling cascade clacineurin-NFATc1/c3-PKD3-NFATc4 in the myocardium is proposed for the PCH model. Cardiac valve development is a very dynamic and complex morphogenetic process involving signals from the myocardium to the endocardium. For example at E9 in heart valve morphogenesis in mice, NFAT is required to repress VEGF expression in the myocardium. However later, at E11, a second wave of calcineurin/NFAT signaling is required in the endocardium, adjacent to the earlier myocardial site of NFAT action, to direct valvular elongation and refinement and is required for valve interstitial cell development (Chang et al., 2004). A conserved role for the second wave of Nfatc1 for VICs is recently also discovered in zebrafish (Gunawan et al., 2020). Our findings propose that a serine/threonine protein kinase (PRKD2) acts synergistically with a serine/threonine phosphatase (calcineurin) pathway to fine-tune heart development and function both in the myocardium and the endocardium plausibly also in several stages.

Moreover, *PRKD2* expression has been correlated with key genes involved in Notch pathway in newly diagnosed acute myeloid leukemia (AML) patients (Liu et al., 2019) and there is evidence that PRKD2 promotes proliferation and chemo-resistance of human AML cell lines through a mechanisms involving Notch activity (Liu et al., 2019). Our observation that *notch1b*, *bmp4*, and the flow responsive transcriptional factor *klf2a* (factors that have been implicated in AV valve formation) (Kalogirou et al., 2014) are detected throughout the hearts of *s411* mutants – these factors do not localize to the AV region – reinforces the notion that PRKD2 sits at a nodal point controlling a number of signaling pathways that regulate AV valve formation. In addition, another study has recently revealed a PRKD1-HDAC4/5-TBX5 regulatory pathway via PRKD1 relief of HDAC4/5-mediated post-translational suppression of TBX5 transcriptional activity (Ghosh et al., 2019). In our study, from an unbiased initial observation that a polymorphism in humans is expected to disturb a TBX5 binding site, we identified Tbx5 elements in the zebrafish promoter of *prkd2* and then verified downregulation of *prkd2* expression upon injection of a well-characterized *tbx5* morpholino. The interaction of PRKD2 with TBX5 suggests that prkd2 is also regulated in the myocardium, a novel regulatory interaction in heart development, which warrants further investigation of the underlying mechanism.

The origin of congenital heart defects has been linked with the properties of cardiac progenitor cells and the mechanisms of their development. Myocardial specification and differentiation during the initial growth of the heart tube, its elongation and the cardiac looping are well-orchestrated processes. In mammals, early cardiomyocytes that contribute to the formation of the left ventricle, atrioventricular canal and atria arise from a collection of progenitor cells named the first heart field (FHF). Subsequent addition of cardiomyocytes to the early cardiac tube that forms the outflow tract, right ventricle, and inflow region occurs via a second distinct cell lineage termed the second heart field (SHF) (Buckingham et al., 2005; Rochais et al., 2009). Thus, the embryonic heart development occurs by cardiomyocytes deriving from two cell lineages corresponding to the contribution of HFH and SHF. Despite the difference of heart morphology between the two-chamber zebrafish heart and mammalian heart, it has been shown that similar regulatory networks drive the fish cardiac cell fate. In the fish model, FHF is the source of cells for the formation of the primitive heart tube, and cells derived from SHF are added to the heart tube and build structures at the inflow and outflow tract (De Pater et al., 2009; Hami et al., 2011; Musso et al., 2015). The discrete cardiomyocyte differentiation phases of zebrafish heart revealed that the two-cell lineage model is a conserved mechanism.

Studies have shown the critical role of cardiac neural crest cells in regulating the second heart field differentiation and that the absence of neural crest-derived mesenchyme in the pharyngeal region, impacts negatively on heart tube elongation. SHF cells contribute to the outflow tract and the structure and morphology of bulbus arteriosus in zebrafish. Several signaling pathways emerge in regulating this complex morphogenetic process where the heart and endocardium continues to the outflow tract endothelium, which is supported by smooth muscle cells. Pathways that play crucial role in atrioventricular development are emerging to be involved also in shaping the outflow tract. These include the Yap1 klf2a/Notch axis (Duchemin et al., 2019) as well as Tgfβ (Boezio et al., 2020). Based on that, it is not surprising that *prkd2* mutants show defects both in the bulbus arteriosus (initially) and the atrioventricular valve. *prkd2* knockout could cause a similar phenotype in mice as in *s411* mutant zebrafish embryos, but conditional and inducible knockouts would be necessary in order to validate and study the respective murine phenotype.

In zebrafish the Prkd family consists of three members of (*prkd1*, *prkd2*, *prkd3*). *prkd2* is located on chromosome 15, whereas both *prkd1* and *prkd3* are found on chromosome 17. Previous studies implicate zebrafish Prkd1 in the regulation of angiogenesis and lymphangiogenesis during development and as essential for tumor angiogenesis; silencing of *prkd1* results in reduced formation of the intersomitic vessels and parachordal lymphangioblasts and abolished tumor angiogenesis (Hollenbach et al., 2013). Of note, while the overall homology of Prkd1, Prkd2, and Prkd3 is between 60–70%, their catalytic domains show 91% amino acid similarity. However, our identification of an inactivating Prkd2-T757A substitution that drives the *s411* mutant phenotype, argues that the Prkd family enzymes play non-redundant roles during zebrafish development. Finally, it also validates the unbiased forward genetic screen approach to identify important functional residues in proteins with key biological activity.

## MATERIALS AND METHODS

### Zebrafish maintenance and breeding

Zebrafish embryos were raised under standard laboratory conditions at 28°C on a 14/10 h day/night cycle according to Aleström et al. (2020). The following transgenic lines were used: *Tg(kdrl:EGFP)^s843^* (endothelia/endocardial) (Jin et al., 2005), *Tg(myl7:DsRed)^s879^* (myocardium) (Chi et al., 2008) and *Tg(Tp1:mCherry)* (Notch-responsive cells) (Ninov et al., 2012). *s411* is identified in (Beis et al., 2005). Embryos were raised up to 120 hpf. Therefore, these experiments are not animal experiments and do not fall under the protection guidelines of the directive 2010/63/EU revising directive 86/609/EEC on the protection of animals used for scientific purposes as adopted on 22 September 2010. Genotypic and adult handling of animals experimentations were approved from the Bioethics and Animal committees of BRFAA and the Veterinary department of Attica region (number 247916, 08/04/20) for facility EL 25 BIOexp 03. Embryos and larvae were anaesthetized for imaging by incubation in tricaine methanesulfonate, MS-222 (0.003%).

### Genetic mapping, mutation detection

For the accurate physical positioning of the mutation, highly annotated zebrafish genetic maps (MGH, LN54, T51) and known microsatellite markers were used. Genetic lineage analysis facilitating markers of 30cM distance throughout the zebrafish genome initially mapped *s411* on chromosome 15 (LG-linkage group15). Polymorphism marker linkage analysis on 865 embryos, restricted *s411* mutation between the markers z6895 (28 recombinants out of 865 mutant embryos) and z51478 (23 recombinants out of 865 mutant embryos), while further fine-mapping located the mutation between markers on the overlapping BACs: CU062627 (1/28 recombinants) and BX924136 3/23 recombinants). Accordingly to the annotated physical map, all the candidate genes in this area were tested (*prkd2*, *gpr184*, *5 s rRNA*, *urgcp* and *dscam-like*). DNA from s411 and wild-type pooled embryos was sequenced of the open reading frames in the region verified a unique A to G mutation resulting to a T757A substitution.

### Morpholino microinjection knockdown

Wild-type zebrafish embryos were injected at the one-cell stage with a splice blocking anti-*prkd2* morpholino, designed to block the splicing of exon 11. cDNA sequencing of injected embryos verified the prediction and revealed that maintain exon 11 introduces a premature stop codon at amino acid 569. anti-*prkd2* morpholino sequence is 5′-ATGTCAAGTTCACTCACTCACCACA-3′. anti-*tbx5a* morpholino sequence is 5′-TTCACTGTCCGCCATGTCGGAGAG-3′ (Gene Tools, LLC).

### Immunohistochemistry

The following primary antibodies were used at the indicated dilution: mouse monoclonal antibody zn5 at 1:10 (ZIRC), eln2 at 1:100 (gift from Professor Frederick Keeley) (Miao et al., 2007). Wild-type and mutant zebrafish embryos at different time-stage (48, 72 and 96 hpf) were fixed overnight at 4°C or for 2 h at room temperature in 4% paraformaldehyde. Wholemount antibody staining was carried out in PBT (4% BSA, 0,3% Triton X in PBS, pH 7.3) overnight at 4°C. Stained embryos were embedded in 4% agarose/PBS (Sigma-Aldrich, A9539-500G) and cut into 120–150 μm sections with a Leica VT1000S vibratome. Sections were covered by the antifading mounting medium, Vectashield (cat. no. H-1000, Vector). Sections were incubated overnight with rhodamine phalloidin (Molecular Probes) at 1:500 in PBDT (PBS, 0.1% Tween, 1% DMSO) for filamentous actin staining.

### RNA extraction and quantitative real-time PCR

Larvae were collected at the stage of 56 hpf, euthanized, transferred in 2 ml tube containing 300 μl TRI Reagent (Sigma-Aldrich, T9424) and homogenized. Total RNA was extracted from the homogenized samples and reverse transcribed into cDNA using PrimeScript RT reagent kit (TaKaRa RR037a) according to the manufacturers' protocols, using 500 ng RNA starting material per cDNA synthesis reaction. qPCR reactions were analyzed on a Roche Lightcycler 96 (Roche Life Science) using KAPA SYBR FAST (Sigma-Aldrich, KK4611). Cycling conditions were as follows: 2 min at 50°C and 10 min at 95°C followed by two-step PCR for 40 cycles of 15 s at 95°C and 60 s at 60°C. Relative expression of *prkd2* was normalized to the average of the stably-expressed reference genes *act2b* and *ef1a*, and calculated 2^−ΔΔCt^ values are presented. The primers used are:

*actin2b :* Fw: 5′-CGAGCTGTCTTCCCATCCA-3′ Rev: 5′-TCACCAACGTAGCTGTCTTTCTG-3′

*prkd2 :* Fw: 5′-GGACTCTTCAGACAAGGGCTTC-3′ Rev: 5′-CCGCACTTCCAGGATTTCCG-3′

*elf1a :* Fw: 5′-TCTCTACCTACCCTCCTCTTGGTC Rev: 5′-TTGGTCTTGGCAGCCTTCTGTG-3′.

### Confocal microscopy

Imaging was performed using Leica STP6000 and Leica TCS SP5 inverted confocal microscope. The images were captured and analyzed with the LAS AF software.

### *In situ* hybridization

Wholemount RNA *in situ* hybridization (ISH) using *notch1b*, *bmp4* and *klf2a* antisense probe was performed in embryos at 72 hpf, according to The Zebrafish Book (Westerfield, 2000).

### Plasmid generation

Mutant forms of human PRKD harboring single residue substitutions were generated using the QuikChange mutagenesis system (Agilent Technologies) and then validated by sequencing. Expression vectors were introduced into HEK293 cells [maintained in Dulbecco's modified Eagle's medium (DMEM) with 10% fetal bovine serum (FBS)] using the Effectene transfection reagent (Qiagen) according to the manufacturer's instructions. After 24 h, cells were lysed in RIPA buffer containing 1 mM sodium orthovanadate, 10 μg ml^−1^ aprotinin, 10 μg ml^−1^ leupeptin, 10 μg ml^−1^ benzamidine, 0.5 mM phenylmethylsulfonyl fluoride, 5 μM pepstatin A, and 0.1 μM calyculin. Cell lysates were used for *in vitro* kinase assays with a recombinant human CREB-maltose binding protein fusion construct (from Biosource) included as substrate according to methods described previously. Immunoblot analysis to track PRKD autophosphorylation and heterologous substrate phosphorylation was performed with antibodies from Cell Signaling Technology according to standard methods and as described previously (Guo et al., 2011).

## Supplementary Material

Supplementary information

## References

[BIO058542C1] Aleström, P., D'Angelo, L., Midtlyng, P. J., Schorderet, D. F., Schulte-Merker, S., Sohm, F. and Warner, S. (2020). Zebrafish: Housing and husbandry recommendations. *Lab. Anim.* 54, 213-224. 10.1177/0023677219869037 31510859 PMC7301644

[BIO058542C2] Alter, S., Zimmer, A. D., Park, M., Gong, J., Caliebe, A., Fölster-Holst, R., Torrelo, A., Colmenero, I., Steinber,g, S. F. and Fischer, J. (2020). Telangiectasia-ectodermal dysplasia-brachydactyly-cardiac anomaly syndrome is caused by de novo mutations in protein kinase D1. *J. Med. Genet.* jmedgenet-2019-106564. 10.1136/jmedgenet-2019-106564 32817298

[BIO058542C3] Bartman, T., Walsh, E. C., Wen, K. K., McKane, M., Ren, J., Alexander, J., Rubenstein, P. A. and Stainier, D. Y. (2004). Early myocardial function affects endocardial cushion development in zebrafish. *PLoS Biol.* 2, E129. 10.1371/journal.pbio.0020129 15138499 PMC406391

[BIO058542C4] Begemann, G. and Ingham, P. W. (2000). Developmental regulation of Tbx5 in zebrafish embryogenesis. *Mech. Dev.* 90, 299-304. 10.1016/S0925-4773(99)00246-4 10640716

[BIO058542C5] Beis, D., Bartman, T., Jin, S. W., Scott, I. C., D'Amico, L. A., Ober, E. A., Verkade, H., and J, F., Field, H. A., Wehman, A. et al. (2005). Genetic and cellular analyses of zebrafish atrioventricular cushion and valve development. *Development* 132, 4193-4204. 10.1242/dev.01970 16107477

[BIO058542C6] Beis, D., Kalogirou, S. and Tsigkas, N. (2015). Insights into Heart Development and Regeneration. In *Introduction to Translational Cardiovascular Research* (ed. D. V. Cokkinos). Springer; 2015: Chapter 2 (p.17-30). 10.1007/978-3-319-08798-6_2

[BIO058542C7] Boezio, G. L. M., Bensimo-Brito, A., Piesker, J., Guenther, S., Helker, C. S. M. and Stainier, D. Y. R. (2020). Endothelial TGF-β signaling instructs smooth muscle development in the cardiac outflow tract. *eLife*. 9, e57603. 10.7554/eLife.57603 32990594 PMC7524555

[BIO058542C8] Bournele, D. and Beis, D. (2016). Zebrafish models of cardiovascular disease. *Heart Fail. Rev.* 21, 803-813. 10.1007/s10741-016-9579-y 27503203

[BIO058542C9] Buckingham, M., Meilhac, S. and Zaffran, S. (2005). Building the mammalian heart from two sources of myocardial cells. *Nat. Rev. Genet.* 6, 826-835. 10.1038/nrg1710 16304598

[BIO058542C10] Chang, C.-P., Neilson, J. R., Bayle, J. H., Gestwicki, J. E., Kuo, A., Stankunas, K., Graef, I. A. and Crabtree, G. R. (2004). A field of myocardial-endocardial NFAT signaling underlies heart valve morphogenesis. *Cell* 118, 649-663. 10.1016/j.cell.2004.08.010 15339668

[BIO058542C11] Chapman, D. L., Garvey, N., Hancock, S., Alexiou, M., Agulnik, S. I., Gibson-Brown, J. J., Cebra-Thomas, J., Bollag, R. J., Silver, L. M. and Papaioannou, V. E. (1996). Expression of the T-box family genes, Tbx1–Tbx5, during early mouse development. *Dev. Dyn.* 206, 379-390. 10.1002/(SICI)1097-0177(199608)206:4<379::AID-AJA4>3.0.CO;2-F 8853987

[BIO058542C12] Chen, J., Deng, F., Li, J. and Wang, Q. J. (2008). Selective binding of phorbol esters and diacylglycerol by individual C1 domains of the PKD family. *Biochem. J.* 411, 333-342. 10.1042/BJ20071334 18076381

[BIO058542C13] Chi, N. C., Shaw, R. M., De Val, S., Kang, G., Jan, L. Y., Black, B. L. and Stainier, D. Y. (2008). Foxn4 directly regulates tbx2b expression and atrioventricular canal formation. *Genes Dev.* 22, 734-739. 10.1101/gad.1629408 18347092 PMC2275426

[BIO058542C14] de la Pompa, J. L., Timmerman, L. A., Takimoto, H., Yoshida, H., Elia, A. J., Samper E, P., Wakeham A, J., Marengere, L., Langille, B. L. and Crabtree GR, M. T. (1998). Role of the NF-ATc transcription factor in morphogenesis of cardiac valves and septum. *Nature* 392, 182-186. 10.1038/32419 9515963

[BIO058542C15] De Pater, E., Clijsters, L., Marques, S. R., Lin, Y. F., Garavito-Aguilar, Z. V., Yelon, D. and Bakkers, J. (2009). Distinct phases of cardiomyocyte differentiation regulate growth of the zebrafish heart. *Development* 136, 1633-1641. 10.1242/dev.030924 19395641 PMC2673760

[BIO058542C16] Duchemin, A. L., Vignes, H. and Vermot, J. (2019). Mechanically activated Piezo channels modulate outflow tract valve development through Yap1 and Klf2-Notch signaling axis. *eLife*. 8, e44706. 10.7554/eLife.44706 31524599 PMC6779468

[BIO058542C17] Fielitz, J., Kim, M. S., Shelton, J. M., Qi, X., Hill, J. A., Richardson, J. A., Bassel-Duby, R. and Olson, E. N. (2008). Requirement of protein kinase D1 for pathological cardiac remodeling. *Proc. Natl. Acad. Sci. USA* 105, 3059-3063. 10.1073/pnas.0712265105 18287012 PMC2268584

[BIO058542C18] Garrity, D. M., Childs, S. and Fishman, M. C. (2002). The heartstrings mutation in zebrafish causes heart/fin Tbx5 deficiency syndrome. *Development* 129, 4635-4645. 12223419 10.1242/dev.129.19.4635

[BIO058542C19] Ghosh, T. K., Aparicio-Sanchez, J., Buxton, S. and Brook, J. D. (2019). HDAC4 and 5 repression of TBX5 is relieved by protein kinase D1. *Sci. Rep.* 9, 1-10. 10.1038/s41598-018-37186-2 31784580 PMC6884511

[BIO058542C20] Giardoglou, P. and Beis, D. (2019). On Zebrafish disease models and matters of the heart. *Biomedicines* 7, 15. 10.3390/biomedicines7010015 PMC6466020 30823496

[BIO058542C21] Gibson-Brown, J. J., Agulnik, S., Silver, L. M. and Papaioannou, V. E. (1998). Expression of T-box genes Tbx2–Tbx5 during chick organogenesis. *Mech. Dev.* 74, 165-169. 10.1016/S0925-4773(98)00056-2 9651516

[BIO058542C22] Gunawan, F., Gentile, A., Gauvrit, S., Stainier, D. and Bensimon-Brito, A. (2020). Nfatc1 promotes interstitial cell formation during cardiac valve development in Zebrafish. *Circ. Res.* 126, 968-984. 10.1161/CIRCRESAHA.119.315992 32070236

[BIO058542C23] Guo, J., Gertsberg, Z., Ozgen, N., Sabri, A. and Steinberg, S. F. (2011). Protein kinase D isoforms are activated in an agonist-specific manner in cardiomyocytes. *J. Biol. Chem.* 286, 6500-6509. 10.1074/jbc.M110.208058 21156805 PMC3057797

[BIO058542C24] Hami, D., Grimes, A. C., Tsai, H. J. and Kirby, M. L. (2011). Zebrafish cardiac development requires a conserved secondary heart field. *Development* 138, 2389-2398. 10.1242/dev.061473 21558385 PMC3091499

[BIO058542C25] Hayashi, A., Seki, N., Hattori, A., Kozuma, S. and Saito, T. (1999). PKCν, a new member of the protein kinase C family, composes a fourth subfamily with PKCμ. *Biochim. Biophys. Acta* 1450, 99-106. 10.1016/S0167-4889(99)00040-3 10231560

[BIO058542C26] Hoffman, J. I. and Kaplan, S. (2002). The incidence of congenital heart disease. *J. Am. Coll. Cardiol.* 39, 1890-1900. 10.1016/S0735-1097(02)01886-7 12084585

[BIO058542C27] Hollenbach, M., Stoll, S. J., Jörgens, K., Seufferlein, T. and Kroll, J. (2013). Different regulation of physiological and tumor angiogenesis in Zebrafish by protein kinase D1 (PKD1). *PLoS ONE* 8, e68033. 10.1371/journal.pone.0068033 23874489 PMC3706615

[BIO058542C28] Iglesias, T. and Rozengurt, E. (1998). Protein kinase D activation by mutations within its pleckstrin homology domain. *J. Biol. Chem.* 273, 410-416. 10.1074/jbc.273.1.410 9417097

[BIO058542C29] Iglesias, T. and Rozengurt, E. (1999). Protein kinase D activation by deletion of its cysteine-rich motifs. *FEBS Lett.* 454, 53-56. 10.1016/S0014-5793(99)00772-3 10413094

[BIO058542C30] Iglesias, T., Matthews, S. and Rozengurt, E. (1998). Dissimilar phorbol ester binding properties of the individual cysteine-rich motifs of protein kinase D. *FEBS Lett.* 437, 19-23. 10.1016/S0014-5793(98)01189-2 9804164

[BIO058542C31] Jin, S. W., Beis, D., Mitchell, T., Chen, J. N. and Stainier, D. Y. (2005). Cellular and molecular analyses of vascular tube and lumen formation in zebrafish. *Development* 132, 5199-5209. 10.1242/dev.02087 16251212

[BIO058542C32] Johnson, K. R., Nicodemus-Johnson, J., Spindler, M. J. and Carnegie, G. K. (2015). Genome-wide gene expression analysis shows AKAP13-mediated PKD1 signaling regulates the transcriptional response to cardiac hypertrophy. *PLoS ONE* 10, e0132474. 10.1371/journal.pone.0132474 26192751 PMC4508115

[BIO058542C33] Just, S., Berger, I. M., Meder, B., Backs, J., Keller, A., Marquar, S., Frese, K., Patzel, E., Rauch, G. J., Tübingen 2000 Screen Consortium, Katus, H. A. et al. (2011). Protein kinase D2 controls cardiac valve formation in zebrafish by regulating histone deacetylase 5 activity. *Circulation* 124, 324-334. 10.1161/CIRCULATIONAHA.110.003301 21730303

[BIO058542C34] Kalogirou, S., Malissovas, N., Moro, E., Argenton, F., Stainier, D. Y. R. and Beis, D. (2014). Intracardiac flow dynamics regulate atrioventricular valve morphogenesis. *Cardiovasc. Res.* 104, 49-60. 10.1093/cvr/cvu186 25100766 PMC4271066

[BIO058542C35] Kim, M.-S., Fielitz, J., McAnally, J., Shelton, J. M., Lemon, D. D., McKinsey, T. A., Richardson, J. A., Bassel-Duby, R. and Olson, E. N. (2008). Protein Kinase D1 Stimulates MEF2 Activity in Skeletal Muscle and Enhances Muscle Performance. *Mol. Cell. Biol.* 28, 3600-3609. 10.1128/MCB.00189-08 18378694 PMC2423294

[BIO058542C36] Kreft, L., Soete, A., Hulpiau, P., Botzki, A., Saeys, Y. and De Bleser, P. (2017). ConTra v3: a tool to identify transcription factor binding sites across species, update 2017. *Nucleic Acids Res.* 45, W490-W494. 10.1093/nar/gkx376 28472390 PMC5570180

[BIO058542C37] Li, C., Li, J., Cai, X., Sun, H., Jiao, J., Bai, T., Zhou, X. W., Chen, X., Gill, D. L. and Tang, X. D. (2011). Protein kinase D3 is a pivotal activator of pathological cardiac hypertrophy by selectively increasing the expression of hypertrophic transcription factors. *J. Biol. Chem.* 286, 40782-40791. 10.1074/jbc.M111.263046 21971046 PMC3220477

[BIO058542C38] Liu, Q., Li, W., Zhou, Y., Jian, J., Han, S., Liu, C., Li, W., Zhu, X., Ma, D., Ji, M. et al. (2019). Prkd2 promotes progression and chemoresistance of aml via regulating notch1 pathway. *Onco Targets Ther.* 12, 10931-10941. 10.2147/OTT.S233234 31849496 PMC6913764

[BIO058542C39] Manning, G., Whyte, D. B., Martinez, R., Hunter, T. and Sudarsanam, S. (2002). The protein kinase complement of the human genome. *Science* 298, 1912-1934. 10.1126/science.1075762 12471243

[BIO058542C40] Miao, M., Bruce, A. E., Bhanji, T., Davis, E. C. and Keeley, F. W. (2007). Differential expression of two tropoelastin genes in zebrafish. *Matrix Biol.* 26, 115-124. 10.1016/j.matbio.2006.09.011 17112714

[BIO058542C41] Musso, G., Mosimann, C., Panáková, D., Burger, A., Zhou, Y., Zon, L. I. and MacRae, C. A. (2015). Generating and evaluating a ranked candidate gene list for potential vertebrate heart field regulators. *Genomics Data* 6, 199-201. 10.1016/j.gdata.2015.09.015 26697374 PMC4664750

[BIO058542C42] Ninov, N., Borius, M. and Stainier, D. Y. R. (2012). Different levels of Notch signaling regulate quiescence, renewal and differentiation in pancreatic endocrine progenitors. *Development* 139, 1557-1567. 10.1242/dev.076000 22492351 PMC3317964

[BIO058542C43] Oster, H., Abraham, D. and Leitges, M. (2006). Expression of the protein kinase D (PKD) family during mouse embryogenesis. *Gene Expression Patterns* 6, 400-408. 10.1016/j.modgep.2005.09.006 16377259

[BIO058542C44] Piscuoglio, S., Fusco, N., Ng, C. K., Martelotto, L. G., da Cruz Paula, A., Katabi N, R., Skálová A, B. P., Weinreb, I. and Weigelt B, R.-F. J. (2016). Lack of PRKD2 and PRKD3 kinase domain somatic mutations in PRKD1 wild-type classic polymorphous low-grade adenocarcinomas of the salivary gland. *Histopathology* 68, 1055-1062. 10.1111/his.12883 26426580 PMC5600617

[BIO058542C45] Prasad, A. M. and Inesi, G. (2009). Effects of thapsigargin and phenylephrine on calcineurin and protein kinase C signaling functions in cardiac myocytes. *Am. J. Physiol. Cell Physiol.* 296, 992-1002. 10.1152/ajpcell.00594.2008 PMC2681384 19244478

[BIO058542C46] Rey, O. and Rozengurt, E. (2001). Protein kinase D interacts with golgi via its cysteine-rich domain. *Biochem. Biophys. Res. Commun.* 287, 21-26. 10.1006/bbrc.2001.5530 11549247

[BIO058542C47] Rochais, F., Dandonneau, M., Mesbah, K., Jarry, T., Mattei, M. G. and Kelly, R. G. (2009). Hes1 Is expressed in the second heart field and is required for outflow tract development. *PLoS ONE* 4, e6267. 10.1371/journal.pone.0006267 19609448 PMC2707624

[BIO058542C48] Rozengurt, E., Rey, O. and Waldron, R. T. (2005). Protein kinase D signaling. *J. Biol. Chem.* 280, 13205-13208. 10.1074/jbc.R500002200 15701647

[BIO058542C49] Sehnert, A. J., Huq, A., Weinstein, B. M., Walker, C., Fishman, M. and Stainier, D. Y. (2002). Cardiac troponin T is essential in sarcomere assembly and cardiac contractility. *Nat. Genet.* 31, 106-110. 10.1038/ng875 11967535

[BIO058542C50] Shaheen, R., Al Hashem, A., Alghamdi, M. H., Seidahmad, M. Z., Wakil, S. M., Dagriri, K., Keavney, B., Goodship, J., Alyousif, S., Al-Habshan, F. M. et al. (2015). Positional mapping of PRKD1, NRP1 and PRDM1 as novel candidate disease genes in truncus arteriosus. *J. Med. Genet.* 52, 322-329. 10.1136/jmedgenet-2015-102992 25713110

[BIO058542C51] Sifrim, A., Hitz, M.-P., Wilsdon, A., Breckpot, J., Al Turki, S. H., Thienpont, B., McRae, J., Fitzgerald, T. W., Singh, T., Swaminathan, G. J. et al. (2016). Distinct genetic architectures for syndromic and nonsyndromic congenital heart defects identified by exome sequencing. *Nat. Genet.* 48, 1060-1065. 10.1038/ng.3627 27479907 PMC5988037

[BIO058542C52] Speliotes, E. K., Willer, C. J., Berndt, S. I., Monda, K. L., Thorleifsson, G., Jackson, A. U., Lango Allen, H., Lindgren, C. M., Luan, J., Mägi, R. et al. (2010). Association analyses of 249,796 individuals reveal 18 new loci associated with body mass index. *Nat. Genet.* 42, 937-948. 10.1038/ng.686 20935630 PMC3014648

[BIO058542C53] Stainier, D. Y. R., Beis, D., Jungblut, B. and Bartman, T. (2002). Endocardial cushion formation in zebrafish. *Cold Spring Harbor Symp. Quant. Biol.* 67, 49-56. 10.1101/sqb.2002.67.49 12858523

[BIO058542C54] Sturany, S., Van Lint, J., Muller, F., Wilda, M., Hameister, H., Hocker, M., Brey, A., Gern, U., Vandenheede, J., Gress, T. et al. (2001). Molecular cloning and characterization of the human protein kinase D2: A novel member of the protein kinase D family of serine threonine kinases. *J. Biol. Chem.* 276, 3310-3318. 10.1074/jbc.M008719200 11062248

[BIO058542C55] Sugi, Y., Yamamura, H., Okagawa, H. and Markwald, R. R. (2004). Bone morphogenetic protein-2 can mediate myocardial regulation of atrioventricular cushion mesenchymal cell formation in mice. *Dev. Biol.* 269, 505-518. 10.1016/j.ydbio.2004.01.045 15110716

[BIO058542C56] Timmerman, L. A., Grego-Bessa, J., Raya, A., Bertrán, E., Pérez-Pomares, J. M., Díez, J., Aranda, S., Palomo, S., McCormick, F., Izpisúa-Belmonte, J. C. et al. (2004). Notch promotes epithelial-mesenchymal transition during cardiac development and oncogenic transformation. *Genes Dev.* 18, 99-115. 10.1101/gad.276304 14701881 PMC314285

[BIO058542C57] Tsybouleva, N., Zhang, L., Chen, S., Patel, R., Lutucuta, S., Nemoto, S., DeFreitas, G., Entman, M., Carabello, B. A., Roberts, R. et al. (2004). Aldosterone, through novel signaling proteins, is a fundamental molecular bridge between the genetic defect and the cardiac phenotype of hypertrophic cardiomyopathy. *Circulation* 109, 1284-1291. 10.1161/01.CIR.0000121426.43044.2B 14993121 PMC2779533

[BIO058542C58] Valverde, A. M., Sinnett-Smith, J., Van Lint, J. and Rozengurt, E. (1994). Molecular cloning and characterization of protein kinase D: a target for diacylglycerol and phorbol esters with a distinctive catalytic domain. *Proc. Natl. Acad. Sci. U.S.A.* 91, 8572-8576. 10.1073/pnas.91.18.8572 8078925 PMC44648

[BIO058542C59] Vermot, J., Forouhar, A. S., Liebling, M., Wu, D., Plummer, D., Gharib, M. and Fraser, S. E. (2009). Reversing blood flows act through klf2a to ensure normal valvulogenesis in the developing heart. *PLoS Biol.* 7, e1000246. 10.1371/journal.pbio.1000246 19924233 PMC2773122

[BIO058542C60] Waldron, R. T., Iglesias, T. and Rozengurt, E. (1999). The pleckstrin homology domain of protein kinase D interacts preferentially with the η isoform of protein kinase C. *J. Biol. Chem.* 274, 9224-9230. 10.1074/jbc.274.14.9224 10092595

[BIO058542C61] Walsh, E. C. and Stainier, D. Y. R. (2001). UDP-Glucose dehydrogenase required for cardiac valve formation in Zebrafish. *Science* 293, 1670-1673. 10.1126/science.293.5535.1670 11533493

[BIO058542C62] Wei, N., Chu, E., Wu, S., Wipf, P. and Schmitz, J. C. (2015). The cytotoxic effects of regorafenib in combination with protein kinase D inhibition in human colorectal cancer cells. *Oncotarget* 6, 4745-4756. 10.18632/oncotarget.2938 25544765 PMC4467112

[BIO058542C63] Westerfield, M. (2000). *‘The Zebrafish Book’: A Guide for the Laboratory use of Zebrafish (Danio rerio)*, 4th edn Eugene: Univ. of Oregon Press.

[BIO058542C64] Yuan, J. and Pandol, S. J. (2016). PKD signaling and pancreatitis. *J. Gastroenterol.* 51, 651-659. 10.1007/s00535-016-1175-3 26879861 PMC4921304

