# Supplementary material for: A zebrafish forward genetic screen identifies an indispensable threonine residue in the kinase domain of PRKD2
Source: Biol Open. 2021 Mar 9;10(3):bio058542. doi: 10.1242/bio.058542 (PMC7969590; doi:10.1242/bio.058542)
Supplement: Supplementary information [file biolopen-10-058542-s1.pdf]

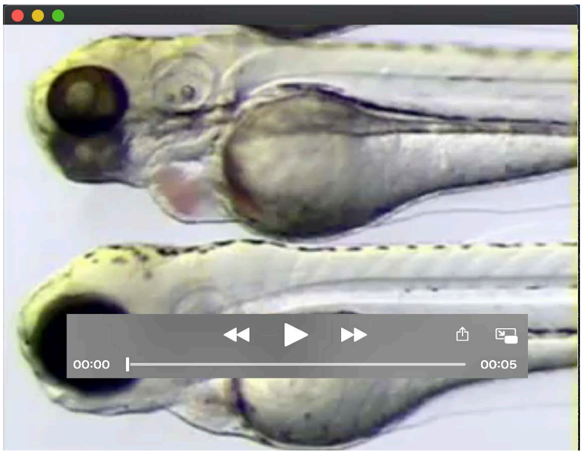

Movie 1. Brightfield video of wild-type (bottom) versus *s411* (top) at 72hpf, showing the outflow tract stenosis at physiological speed.

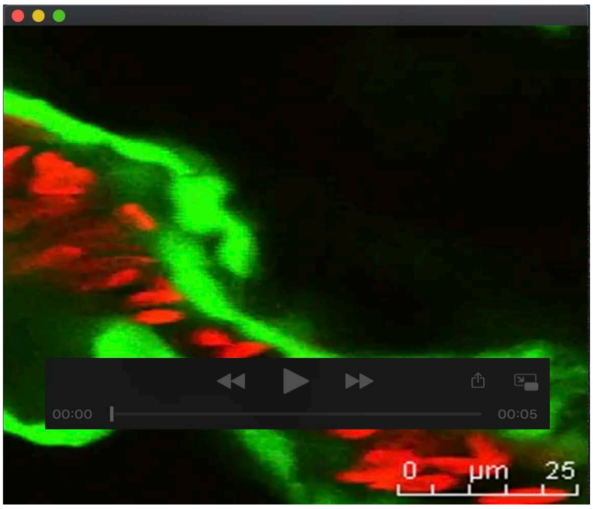

Movie 2. Resonant scanner confocal movie of the outflow tract of wild-type *Tg(kdrl::eGFP);Tg(gata1::DsRed) at 1/10 speed.*

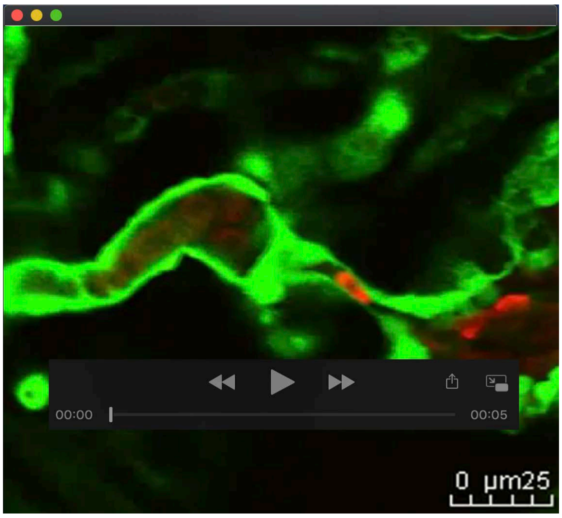

Movie 3. Resonant scanner confocal movie of the outflow tract of *s411 Tg(kdrl::eGFP);Tg(gata1::DsRed) at 1/10 speed.*

Biology Open • Supplementary information
